# Association between Arsenic Suppression of Adipogenesis and Induction of CHOP10 via the Endoplasmic Reticulum Stress Response

**DOI:** 10.1289/ehp.1205731

**Published:** 2012-12-05

**Authors:** Yongyong Hou, Peng Xue, Courtney G. Woods, Xia Wang, Jingqi Fu, Kathy Yarborough, Weidong Qu, Qiang Zhang, Melvin E. Andersen, Jingbo Pi

**Affiliations:** 1 Institute for Chemical Safety Sciences, The Hamner Institutes for Health Sciences, Research Triangle Park, North Carolina, USA; 2 School of Public Health, Fudan University, Shanghai, China

**Keywords:** adipogenesis, arsenic, C/EBP, CHOP10, preadipocytes, type 2 diabetes

## Abstract

Background: There is growing evidence that chronic exposure to inorganic arsenic (iAs) is associated with an increased prevalence of type 2 diabetes (T2D). However, the mechanisms for the diabetogenic effect of iAs are still largely unknown. White adipose tissue (WAT) actively stores and releases energy and maintains lipid and glucose homeostasis.

Objective: We sought to determine the mechanisms of arsenic suppression of adipogenesis.

Methods: The effects and associated mechanisms of iAs and its major metabolites on adipogenesis were determined in 3T3-L1 preadipocytes, mouse adipose-derived stromal-vascular fraction cells (ADSVFCs), and human adipose tissue–derived stem cells (ADSCs).

Results: Exposure of 3T3-L1 preadipocytes to noncytotoxic levels of arsenic, including inorganic arsenite (iAs^3+^, ≤ 5 μM), inorganic arsenate (≤ 20 μM), trivalent monomethylated arsenic (MMA^3+^, ≤ 1 μM), and trivalent dimethylated arsenic (DMA^3+^, ≤ 2 μM) decreased adipogenic hormone-induced adipogenesis in a concentration-dependent manner. In addition, iAs^3+^, MMA^3+^, and DMA^3+^ exhibited a strong inhibitory effect on adipogenesis in primary cultured mouse ADSVFCs and human ADSCs. Time-course studies in 3T3-L1 cells revealed that inhibition of adipogenesis by arsenic occurred in the early stage of terminal adipogenic differentiation and was highly correlated with the induction of C/EBP homologous protein (CHOP10), an endoplasmic reticulum (ER) stress response protein. Induction of CHOP10 by arsenic is associated with reduced DNA-binding activity of CCAAT/enhancer-binding protein β (C/EBPβ), which regulates the transcription of peroxisome proliferator-activated receptor γ and C/EBPα.

Conclusions: Low-level iAs and MMA^3+^ trigger the ER stress response and up-regulate CHOP10, which inhibits C/EBPβ transcriptional activity, thus suppressing adipogenesis. Arsenic-induced dysfunctional adipogenesis may be associated with a reduced capacity of WAT to store lipids and with insulin resistance.

There is growing evidence that chronic exposure to inorganic arsenic (iAs) is associated with an increased prevalence of diabetes mellitus, a metabolic disease attributed to a combination of genetic, lifestyle, and environmental factors (Maull et al. 2012). Although the precise mechanisms for the diabetogenic effect of iAs are still largely undefined, epidemiological studies have demonstrated that type 2 diabetes (T2D) is an important type of diabetes that is associated with chronic iAs exposure (Andra et al. 2013; Coronado-González et al. 2007; Islam et al. 2012; Maull et al. 2012; Tseng 2004; Tseng et al. 2000). It is clear that insulin resistance plays an early pathogenic role in the development of T2D, and defects in insulin secretion by pancreatic β cells are instrumental in the progression to hyperglycemia (Lowell and Shulman 2005). Experimental studies have indicated that iAs and its trivalent methylated metabolites impair insulin signaling, thus suppressing insulin-dependent glucose uptake (Hill et al. 2009; Izquierdo-Vega et al. 2006; Paul et al. 2007; Walton et al. 2004; Xue et al. 2011).

Insulin resistance, a state in which the response to insulin is blunted, may be initiated in multiple tissues, including white adipose tissue (WAT) (Rosen and Spiegelman 2006). WAT stores and releases energy, maintains lipid and glucose homeostasis, and secretes a variety of factors that influence appetite, insulin sensitivity, inflammation, and other pathways of biological and clinical significance (Rosen and Spiegelman 2006). Excess accumulation of WAT, as in obesity, is a risk factor for insulin resistance and T2D. Conversely, defects in adipogenesis in WAT, as in lipodystrophy, that impair the capacity of WAT to expand and store lipid, can also result in insulin resistance and T2D (Garg and Agarwal 2009; Vigouroux et al. 2011). Although previous *in vitro* studies have demonstrated that low levels of inorganic arsenite (iAs^3+^) suppress adipogenic differentiation (Trouba et al. 2000; Wang et al. 2005; Wauson et al. 2002), the molecular mechanisms underlying the inhibitory effect are not completely understood. In addition, there is no report on the adipogenic effect of other arsenicals that humans could be indirectly exposed to, such as methylated arsenicals.

Adipogenesis is a complex process in which multipotent mesenchymal stem cells (MSCs) are first committed to preadipocytes and subsequently converted to mature, spherical adipocytes with lipid accumulation (Rosen and MacDougald 2006; Tontonoz and Spiegelman 2008). Adipogenesis is regulated by a complicated network of transcription factors that coordinate expression of hundreds of proteins responsible for establishing the mature fat-cell phenotype (Rosen and MacDougald 2006). Early in terminal adipogenesis, the CCAAT/enhancer-binding proteins β and δ (C/EBPβ and C/EBPδ) are rapidly induced to express and later activate expression of peroxisome proliferator-activated receptor γ (PPARγ) and C/EBPα. Although the expression of C/EBPβ rises quickly in preadipocytes in response to adipogenic hormones, its DNA-binding activity is initially suppressed through binding with C/EBP homologous protein (CHOP10, also known as C/EBPζ, DDIT-3, or GADD153) (Rosen and MacDougald 2006). The expression of CHOP10 is down-regulated along with the adipogenic hormone challenge, resulting in transactivation of C/EBPβ. Thus, CHOP10 functions as a negative regulator of C/EBPβ transcriptional activity in the early stage of adipogenesis (Darlington et al. 1998). CHOP10 is ubiquitously expressed at very low levels and highly inducible as a result of the unfolded protein response (UPR), an adaptive response to endoplasmic reticulum (ER) stress. Exposure to iAs and its trivalent methylated metabolites results in ER stress and triggers UPR (Naranmandura et al. 2012; Oh et al. 2012), both of which are involved in many aspects of the pathogenesis of diabetes (Cnop et al. 2011; Oyadomari and Mori 2004). Thus, we hypothesized that suppression of adipogenic differentiation by arsenic is associated with the induction of CHOP10 via UPR. In the present study, we found that low-level iAs and its trivalent monomethylated metabolite trigger UPR and up-regulate CHOP10, which suppresses C/EBPβ transcriptional activity and thus impairs adipogenesis. Arsenic-induced dysfunctional adipogenesis may be associated with reduced capacity of WAT to store lipids and with insulin resistance.

## Materials and Methods

*Reagents.* Insulin solution (I9278), 3-isobutyl-1-methylxanthine (IBMX, I7018), dexamethasone (Dex, D1756), indomethacin (I7378), sodium arsenite (38150), sodium arsenate (iAs^5+^, A6756), disodium methyl arsenate (MMA^5+^, PS281), dimethylarsinic acid (DMA^5+^, PS51), and oil-red O (ORO, 75087) were obtained from Sigma (St. Louis, MO, USA). Rosiglitazone maleate was obtained from SmithKline Beecham Pharmaceuticals (London, UK). Thapsigargin (TG, 586005), tunicamycin (TUN, 654380), and brefeldin A (BFA, 203729) were purchased from EMD Chemicals Inc. (Darmstadt, Germany). Culture media, calf serum, fetal bovine serum (FBS), and supplements were purchased from Invitrogen (Life Technologies, Carlsbad, CA, USA). Methylarsine oxide (CH_3_AsO, MMA^3+^) and dimethylthioarsenite [(CH_3_)_2_AsGSH, DMA^3+^] were synthesized by W.R. Cullen (University of British Columbia, Vancouver, Canada), using a method described previously (Cullen et al. 1989; Petrick et al. 2001; Vega et al. 2001). The compounds were preserved in sealed tubes filled with argon gas.

*Animals.* C57BL/6J mice were obtained from Jackson Laboratories (Bar Harbor, ME, USA) (JAX Stock No. 000664). For experiments, mice that were 12–16 weeks of age were used for preadipocyte isolation. Animals were housed in virus-free facilities on a 12-hr light/12-hr dark cycle and were fed standard rodent food. All protocols for animal use were approved by the institutional animal care and use committee of The Hamner Institutes and were in accordance with National Institutes of Health guidelines. The animals were treated humanely and with regard for alleviation of suffering.

*Cell culture and adipogenic differentiation.* 3T3-L1 preadipocytes were obtained from ATCC (American Type Culture Collection, Manassas, VA, USA) and maintained in high-glucose Dulbecco’s modified Eagle’s medium (DMEM) with 100 U/mL penicillin, 100 µg/mL streptomycin, and 10% calf serum. 3T3-L1 cells were differentiated 1 day after they became confluent (designated as day 0) using the DMI protocol (1 μM dexamethasone, 0.5 mM 3-isobutylmethylxanthine, and 1 μg/mL insulin in DMEM with 10% FBS) as detailed in Supplemental Material, Figure S1 (http://dx.doi.org/10.1289/ehp.1205731) and described previously (Hou et al. 2012). All the cells were maintained at 37°C in a 5% carbon dioxide environment.

Mouse adipose-derived stromal-vascular fraction cells (ADSVFCs) were isolated from WAT as described previously (Cannon and Nedergaard 2001) and cultured in high-glucose DMEM supplemented with 10% FBS, 8.3 mM l-glutamine, 100 U/ml penicillin, and 100 μg/mL streptomycin. Human adipose tissue–derived stem cells (ADSCs) were obtained from Invitrogen and cultured in the MSC growth media (ATCC) according to the manufacturer’s recommendation. To induce differentiation, confluent mouse ADSVFCs and human ADSCs were treated using the DMIRI protocol (DMI, 1 μM rosiglitazone, and 125 μM indomethacin in DMEM with 10% FBS) as detailed in Supplemental Material, Figure S1 (http://dx.doi.org/10.1289/ehp.1205731) and described previously (Hou et al. 2012). Differentiation of preadipocytes to mature adipocytes was confirmed by ORO staining of lipid vesicles as described previously (Hou et al. 2012).

*Acute cytotoxicity assay*. A minimum of five replicates of 10,000 cells per well were plated in 96-well plates and allowed to adhere to the plate for 24 hr, at which time the media was removed and replaced with fresh media containing varying concentrations of arsenicals. Cells were subsequently incubated for an additional 48 hr, and cell viability was determined by using the Non-Radioactive Cell-Proliferation Assay Kit (Promega, Madison, WI, USA) as detailed previously (Xue et al. 2011).

*Immunofluorescence microscopy.* Fluorescence immunostaining was performed as described previously (Xue et al. 2012) and detailed in Supplemental Material, p. 3 (http://dx.doi.org/10.1289/ehp.1205731).

*C/EBP-luciferase reporter assay.* The Cignal Lenti C/EBP reporter, which expresses a luciferase gene driven by multiple transcription response element (ATTGCGCAAT) repeats, was obtained from SABiosciences (Frederick, MD, USA). Lentiviral transduction of 3T3-L1 cells was performed as described previously (Hou et al. 2012). Cells were selected and maintained in medium containing 1.0 μg/mL of puromycin. The luciferase activity was measured using the Luciferase Reporter Assay System (Promega) according to the manufacturer’s protocol. The luciferase activity was normalized to cell viability.

*Quantitative real-time reverse transcription polymerase chain reaction (RT-qPCR).* The isolation, purification, and quantification of RNA and RT-qPCR were performed as described previously (Xue et al. 2012). The primers described in Supplemental Material, Table S1 (http://dx.doi.org/10.1289/ehp.1205731), were designed by using Primer Express 4 (Applied Biosystems, Carlsbad, CA, USA) and synthesized by Bioneer Inc. (Alameda, CA, USA). Real-time fluorescence detection was performed using an ABI PRISM 7900HT Fast Real-time PCR System (Applied Biosystems), and 18S ribosomal RNA (18S) was used for normalization.

*Western blot analysis.* Isolation of cell lysates and immunoblotting were performed as detailed previously (Hou et al. 2012). Antibodies for C/EBPα (sc-61; 1:500), C/EBPβ (sc-7962; 1:500), CHOP10 (sc-575; 1:500), and glyceraldehyde 3-phosphate dehydrogenase (GAPDH, sc-20357; 1:500) were obtained from Santa Cruz Inc. (Santa Cruz, CA, USA). Antibodies for C/EBPδ (#2318; 1:1000), PPARγ (#2435; 1:1000), eukaryotic initiation factor 2α (elF2α) (#9722; 1:1000), and phosphorylated eIF2α (p-eIF2α, #3597; 1:1000) were purchased from Cell Signaling Technology Inc. (Danvers, MA, USA). Antibodies for β-ACTIN (A1978; 1:2000) were purchased from Sigma-Aldrich Inc. (St. Louis, MO, USA). An antibody for activating transcription factor (ATF) 4 (ab23760; 1:1000) was obtained from Abcam Inc. (Cambridge, MA, USA).

*Statistical analyses.* All statistical analyses were performed using GraphPad Prism 4 (GraphPad Software, San Diego, CA, USA), with *p* < 0.05 considered as significant. More specific indices of statistical significance are indicated in individual figure legends. Data are expressed as means ± SDs. For comparisons between two groups, Student’s *t*-test was performed. For comparisons among groups, one-way or two-way analysis of variance with Bonferroni post hoc testing was performed.

## Results

**Figure 1:**
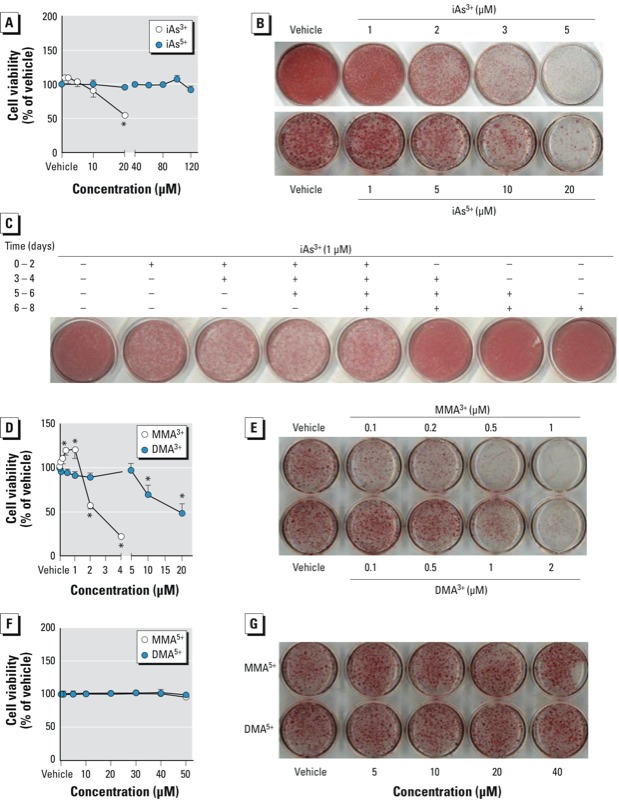
Noncytotoxic levels of iAs suppress adipogenesis in 3T3-L1 preadipocytes. (*A*) Cytotoxicity of iAs^3+^ and iAs^5+^. Cells were exposed to iAs^3+^ and iAs^5+^ in DMEM with 10% FBS for 48 hr, followed by immediate cell viability measurements (*n* = 5–6). (*B*) iAs concentration-dependently inhibits adipogenesis. Cells were differentiated 1 day after 100% confluence (designated as day 0) by replacing growth medium with differentiation medium containing DMI (1 μM dexamethasone, 0.5 mM 3-isobutylmethylxanthine, and 1 μg/mL insulin) in DMEM with 10% FBS for 2 days, followed by an additional 6 days of culture in DMEM with 10% FBS and 1 μg/mL insulin; iAs was added during days 1 and 2 of differentiation; lipid vesicles were stained using ORO. (*C*) Suppression of iAs^3+^ on adipogenesis occurs in the early stage of DMI-induced differentiation. During adipogenesis, iAs^3+^ was added at the indicated periods, and ORO staining was performed on day 8. (*D,F*) Cytotoxicity of methylated arsenicals. Cells were exposed to the arsenicals in DMEM with 10% FBS for 48 hr (*n* = 5–6). (*E,G*) Trivalent, but not pentavalent, methylated arsenicals caused a concentration-dependent inhibition of adipogenesis. MMA^3+^, DMA^3+^, MMA^5+^, and DMA^5+^ were added during days 1 and 2 of differentiation; ORO staining was performed on day 8. Time (days), day of differentiation. *p < 0.05, arsenic-treated vs. vehicle-treated cells.

*Arsenicals suppress adipogenesis in mouse and human preadipocytes*. To assess the association between iAs exposure and adipocyte dysfunction, we examined the effect of various arsenicals on terminal adipogenesis using 3T3-L1 cells. In concordance with previous studies (Trouba et al. 2000; Wang et al. 2005; Wauson et al. 2002), noncytotoxic concentrations of iAs^3+^ exhibited a strong inhibitory effect on adipogenesis induced by the hormonal cocktail DMI (Figure 1A,B). The exposure to iAs^3+^ during the first 2 days of DMI-induced adipogenesis [see Supplemental Material, Figure S1 (http://dx.doi.org/10.1289/ehp.1205731)] substantially attenuated the cellular lipid accumulation by the end of the process in a concentration-dependent manner (Figure 1B). In addition, iAs^3+^-treated cells exhibited a significantly lower expression of many adipogenic genes, including *Cebp*α, *Ppar*γ*1*, *Ppar*γ*2*, retinoid X receptor α (*Rxr*α), adipose differentiation-related protein (*Adrp*), lipoprotein lipase (*Lpl*), complement factor D (adipsin) (*adipsin*), and sterol regulatory element-binding protein 1 (*Srebp1*), along with DMI-induced adipogenesis (see Supplemental Material, Figure S2). Furthermore, at higher concentrations iAs^5+^ also concentration-dependently inhibited DMI-induced adipogenesis (Figure 1B). To determine the timing of iAs^3+^-induced inhibition of adipogenesis, we challenged 3T3-L1 preadipocytes with a low level of iAs^3+^ during different periods of DMI-induced adipogenesis. As shown in Figure 1C, exposure to iAs^3+^ during the first 2 days of DMI-induced adipogenesis was crucial for the inhibition. Without the first 2 days of exposure, later-stage exposure to iAs^3+^ (day 3 through day 8) (Figure 1C) or preexposure to iAs^3+^ (not shown) demonstrated no obvious inhibitory effect on adipogenesis. Thus, the suppression of adipogenesis by iAs^3+^ in 3T3-L1 cells occurs in the first 48 hr of DMI-induced adipogenic differentiation, suggesting that iAs^3+^ may impede early events of adipogenic hormone-triggered adipogenesis.

**Figure 2:**
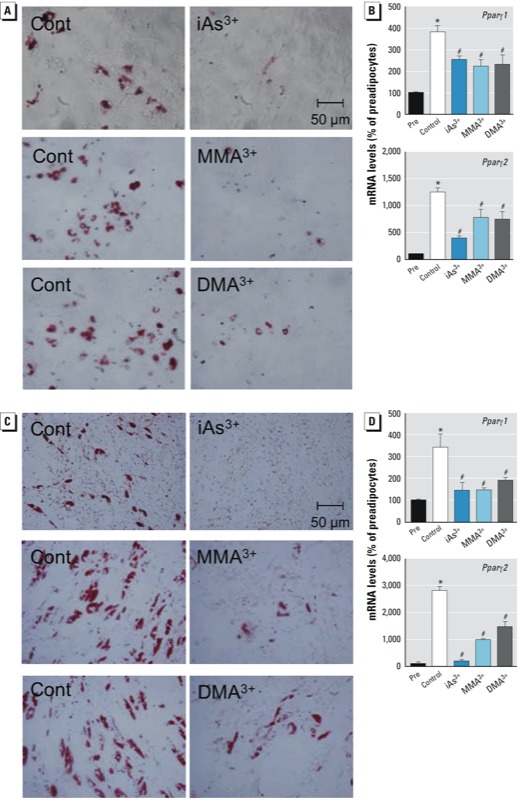
iAs^3+^, MMA^3+^, and DMA^3+^ suppress adipogenesis in mouse ADSVFCs and human ADSCs. Abbreviations: Pre, preadipocytes; Cont, control. Mouse ADSVFCs (*A*,*B*) isolated from C57BL/6J mice and human ADSCs (*C*,*D*) were cultured to 95% confluence and differentiated for 5 days using the DMIRI protocol. iAs^3+^ (5 μM), MMA^3+^ (1 μM for mouse ADSVFCs; 0.2 μM for human ADSCs), and DMA^3+^ (2 μM) were added during days 1 and 2 of differentiation. (*A*,*C*) Photomicrographs of stained cells. After differentiation, cells were stained with ORO to visualize lipid accumulation (20×). (*B*,*D*) mRNA expression of PPARγ (*n* = 3). *p < 0.05, preadipocytes vs. control cells. ^#^*p* < 0.05, arsenic-treated vs. control cells.

To investigate the role of metabolism of iAs in the suppression of arsenic on adipogenesis, the effect of methylated arsenicals on adipogenesis was determined in 3T3-L1 cells. As shown in Figure 1D–G, trivalent methylated arsenicals, including MMA^3+^ and DMA^3+^, but not MMA^5+^ and DMA^5+^, exhibited much more potent inhibitory effects on adipogenesis than iAs. To further ascertain the inhibitory effect of arsenic on adipogenesis, mouse ADSVFCs, which contains MSCs and preadipocytes, were isolated, cultured, and differentiated *in vitro*. As shown in Figure 2A and B, on day 5 of hormonal cocktail DMIRI-induced adipogenesis [see Supplemental Material, Figure S1 (http://dx.doi.org/10.1289/ehp.1205731)], cells treated with iAs^3+^ (5 μM), MMA^3+^ (1 μM), or DMA^3+^ (2 μM) showed substantially reduced levels of lipid accumulation (Figure 2A) and attenuated expression of *Ppar*γ*1* and *Ppar*γ*2* (Figure 2B). In human ADSCs, treatment with iAs^3+^ (5 μM), MMA^3+^ (0.2 μM), or DMA^3+^ (2 μM) also produced a notable inhibition on DMIRI-induced lipid accumulation and *PPAR*γ induction (Figure 2C,D). In addition, MMA^3+^- and DMA^3+^-treated human ADSCs showed reduced expression of adipogenic genes, including *LPL* and cluster of differentiation 36 (*CD36*) (see Supplemental Material, Figure S3). Of note, human ADSCs are sensitive to MMA^3+^-induced cytotoxicity. Thus, a lower noncytotoxic concentration (0.2 μM) was used in adipogenesis studies. Taken together, the findings in 3T3-L1 cells and primary mouse and human cultures demonstrated that iAs and its trivalent methylated metabolites interfere with the early adipogenic event(s) and thus impair terminal adipogenesis.

**Figure 3:**
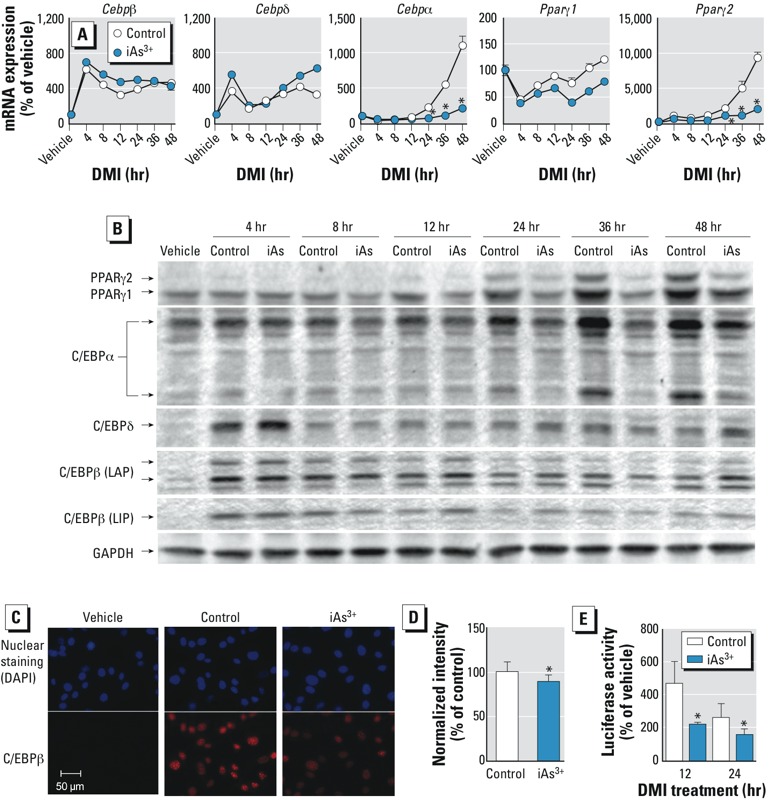
Inhibitory effect of iAs^3+^ on the transcriptional activity of C/EBPβ and expression of PPARγ and C/EBPs during adipogenesis in 3T3-L1 preadipocytes. Abbreviations: C/EBPβ (LAP), C/EBPβ isoform liver-enriched activator protein; C/EBPβ (LIP), C/EBPβ isoform liver-enriched inhibitory protein; Control, cells were differentiated using the DMI protocol for the indicated time; iAs^3+^, cells were treated with iAs^3+^ (5 μM) during DMI treatment; vehicle, cells were maintained in growth medium without DMI. (*A*) mRNA expression of *Cebps* and *Ppar*γ at the same differentiation time (*n* = 3). (*B*) Effects of DMI treatment on protein expression of PPARγ and C/EBPs during adipogenesis. Two isoforms of C/EBPα (42 kDa and 30 kDa) are shown on the blot. (*C*) Representative images of immunostaining of nuclear C/EBPβ after 16-hr DMI treatment. (*D*) Quantification of fluorescence intensity of nuclear C/EBPβ shown in (*C*) (*n* = 4–5). (*E*) Activity of C/EBP-luciferase reporter following DMI treatment in control and iAs^3+^-treated vs. control cells.

*iAs^3+^ inhibits C/EBP*β *transactivation during adipogenesis.* C/EBPβ and C/EBPδ are transiently expressed and play a role at the early stage of adipogenic differentiation by sensing adipogenic stimuli and initiating expression of PPARγ and C/EBPα (Rosen and MacDougald 2006). Upon exposure to adipogenic signals, such as DMI or DMIRI cocktail, C/EBPβ and C/EBPδ are rapidly expressed and transcriptionally regulate the expression of PPARγ and C/EBPα, whereas C/EBPε and CHOP10 serve as negative regulators of PPARγ transcription (Batchvarova et al. 1995; Clarke et al. 1997; Darlington et al. 1998). As C/EBPβ acquires its DNA-binding activity, it becomes localized to centromeres and results in a characteristic “punctuated” pattern in immunofluorescence staining, which is a well-accepted measure of C/EBPβ–DNA-binding activity (Tang and Lane 2000). As shown in Figure 3A,B, iAs^3+^ did not reduce the mRNA and/or protein expression of C/EBPβ and C/EBPδ in the early stage (≤ 48 hr) of adipogenesis. However, the “punctuated” nuclear accumulation of C/EBPβ was reduced by iAs^3+^ after a 16-hr DMI treatment (Figure 3C,D), suggesting that iAs^3+^ directly suppresses centromere accumulation of C/EBPβ and/or interferes with the acquisition of C/EBPβ–DNA-binding activity. In addition, C/EBP–luciferase reporter assay (Figure 3E) and the mRNA expression of C/EBP-target gene *Ppar*γ*2* (Figure 3A) revealed that iAs^3+^ treatment significantly attenuates the transcriptional activity of C/EBPs after ≥ 12 hr of DMI treatment. It appears that the reduced transcriptional activity of C/EBPs caused by iAs^3+^ treatment results from a reduced DNA-binding activity of the C/EBPs, in particular C/EBPβ. Concomitant with the DNA-binding activity of C/EBPβ measured by immunostaining and C/EBP-reporter assay, the expression of PPARγ and C/EBPα in iAs^3+^-treated cells was markedly decreased at a later stage of adipogenesis, particularly after 24 hr of DMI treatment (Figure 3A,B).

**Figure 4:**
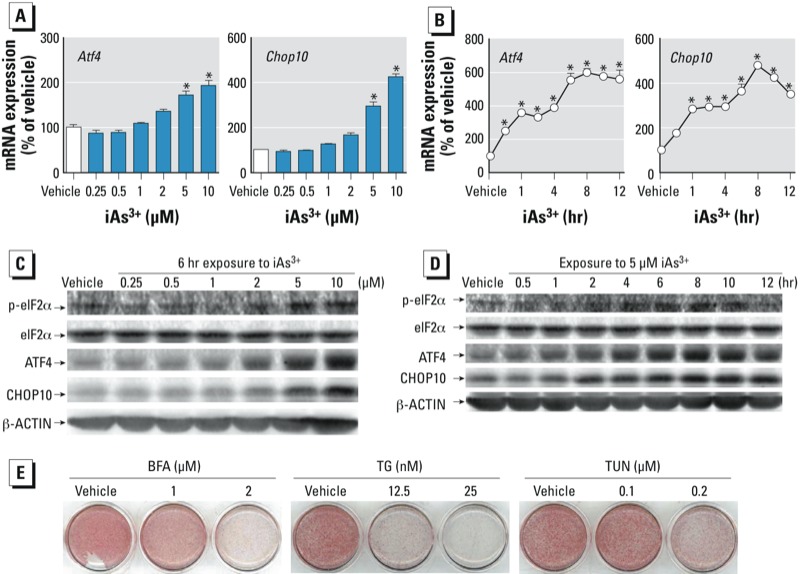
iAs^3+^ activates UPR in 3T3-L1 preadipocytes. Abbreviations: BFA, brefeldin A; TG, thapsigargin; TUN, tunicamycin. (*A*) Six hours of treatment with iAs^3+^ concentration-dependently enhanced mRNA expression of *Atf4* and *Chop10* (*n* = 3). (*B*) Time-course of mRNA expression of *Atf4* and *Chop10* after exposure to 5 μM iAs^3+^ (*n *= 3). (*C,D*) Concentration–response (*C*) and time course (*D*) of protein expression of eIF2α, p-eIF2α, ATF4, and CHOP10 in response to iAs^3+^ treatment. Cells were treated with iAs^3+^ for 6 hr (*C*) or 5 μM iAs^3+^ for the indicated time (*D*) (*n* = 3). (*E*) ER stressors suppress adipogenesis in 3T3-L1 cells. Cells were treated with the stressors at the indicated concentrations during the first 2 days of DMI-induced adipogenesis, followed by ORO staining. *p < 0.05, iAs^3+^-treated vs. vehicle-treated cells.

*iAs^3+^ triggers UPR, which impairs terminal adipogenesis.* To investigate whether UPR, especially CHOP10 induction, is involved in the suppression of terminal adipogenesis by iAs^3+^, the expression of ATF4 and CHOP10 were measured in 3T3-L1 cells. iAs^3+^ concentration- and time-dependently augmented the expression of ATF4 and CHOP10 at mRNA (Figure 4A,B) and protein (Figure 4C,D) levels. In addition, other ER stress responsive genes, such as growth arrest and DNA-damage-inducible 45a (*Gadd45a*), X-box binding protein 1 (*Xbp1*), and spliced *Xbp1* (*sXbp1*) were also significantly induced by iAs^3+^ [see Supplemental Material, Figure S4 (http://dx.doi.org/10.1289/ehp.1205731)], suggesting that iAs^3+^ triggers UPR in the cells. Furthermore, the phosphorylation of eukaryotic initiation factor 2α (p-elF2α) was increased in response to iAs^3+^ treatment, indicating that double-stranded RNA-activated protein kinase (PKR)-like ER kinase (PERK) also plays a role in the response. In 3T3-L1 cells, MMA^3+^ and DMA^3+^ also concentration-dependently increased the mRNA expression of many UPR genes, including *Atf4*, *Chop10*, *Xbp1*, and *sXbp1* (Figures S5 and S6). In addition, MMA^3+^ also concentration- and/or time-dependently increased the mRNA expression of *Gadd45a* (see Supplemental Material, Figure S5) and the protein expression of CHOP10 (see Supplemental Material, Figure S7). These findings suggest that trivalent methylated arsenic may also trigger UPR. In agreement with a previous study (Yu et al. 2009), exposure of 3T3-L1 cells to multiple well-known ER stressors, including BFA, TG, and TUN, at noncytotoxic concentrations resulted in a concentration-dependent suppression of terminal adipogenesis (Figure 4E). Therefore, iAs^3+^ may activate UPR and induce CHOP10 resulting in suppression of adipogenesis.

**Figure 5:**
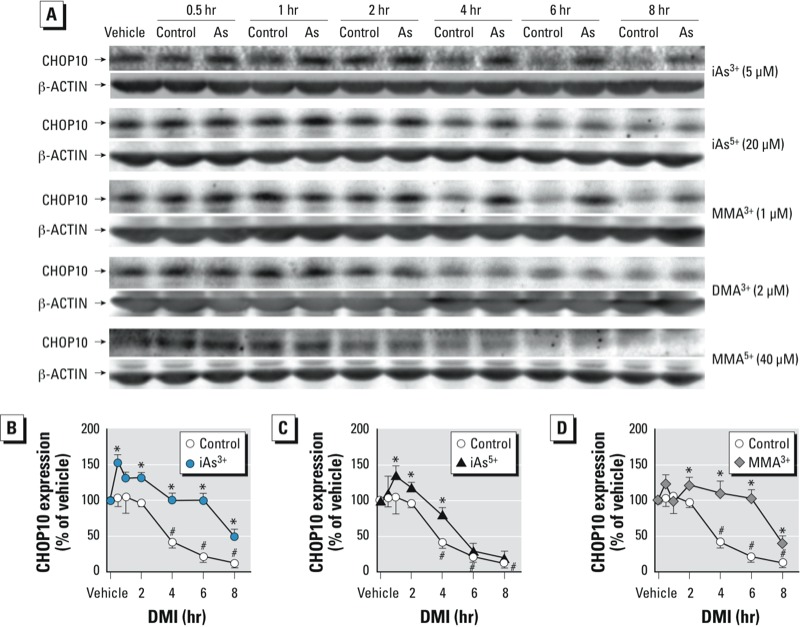
Effects of arsenicals on the DMI-induced reduction of CHOP10 in the early stage of adipogenesis in 3T3-L1 preadipocytes. (*A*) Effects of various arsenicals on the expression of CHOP10 protein in the early stage of adipogenesis. Cells were treated 1 day after confluence with DMI in the absence (control) or presence of arsenicals. Vehicle, growth medium. (*B–D*) Quantification of the protein expression of CHOP10 in response to iAs^3+^, iAs^5+^, or MMA^3+^ exposure in the early stage of adipogenesis (*n* = 3). *p < 0.05, compared with control cells at the same time. ^#^*p* < 0.05, compared with vehicle-treated control cells.

*CHOP10 induction in adipogenic suppression by arsenic.* To further elucidate the involvement of UPR and CHOP10 induction in the arsenic-induced suppression of adipogenesis, the protein expression of CHOP10 was determined during the early stage of DMI-induced adipogenesis. As shown in Figure 5, the expression of CHOP10 in untreated control cells significantly decreased along with the DMI treatment. Comparing with control cells, iAs^3+^ treatment caused significantly attenuated and delayed DMI-induced reduction of CHOP10 protein expression (Figure 5A,B). In agreement with the effects on adipogenesis, MMA^3+^ and iAs^5+^, but not MMA^5+^, also significantly blocked the DMI-induced reduction of CHOP10 protein expression (Figure 5A,B). These findings suggest that the inhibition of terminal adipogenesis by iAs^3+^, MMA^3+^, and iAs^5+^ is, at least in part, due to an inability to decrease CHOP10 in the initiation phase of adipogenesis. DMA^3+^ did not affect DMI-induced reduction of CHOP10 protein expression during the early stage of adipogenesis, suggesting DMA^3+^ impairs adipogenesis by a different mechanism.

## Discussion

Adipogenesis is regulated by a complicated network of transcription factors that coordinate expression of hundreds of proteins responsible for establishing the mature fat-cell phenotype (Rosen and MacDougald 2006). The current model for adipogenesis begins with the increased expression of C/EBPβ, presumably through transcriptional activation by cAMP-response element-binding protein, Kruppel-like factor 4, early growth response protein 2, and nuclear factor E2-related factor 2 (Hou et al. 2012). C/EBPβ then induces expression of PPARγ and C/EBPα, which form a positive feedback loop by activating each other’s expression and play roles at a later stage by inducing and maintaining the expression of adipocyte-specific genes (Rosen and MacDougald 2006; Tontonoz and Spiegelman 2008). Although the expression of C/EBPβ rises quickly in preadipocytes in response to adipogenic hormones, its DNA-binding activity is initially suppressed through binding with CHOP10 (Tang and Lane 2000). Along with adipogenesis, the expression of CHOP10 is down-regulated and results in acquisition of C/EBPβ transcriptional activity (Tang and Lane 2000). Previous studies showed that iAs^3+^ suppresses adipogenesis by inhibiting the expression of PPARγ and C/EBPα or affecting the interaction between PPARγ and RXRα or phosphorylated thymoma viral proto-oncogene (AKT) (Trouba et al. 2000; Wang et al. 2005; Wauson et al. 2002). In the present study, we demonstrated for the first time that iAs^3+^, MMA^3+^, and DMA^3+^ suppress adipogenesis in mouse ADSVFCs and human ADSCs, and we further characterized the inhibitory effect of iAs and its metabolites on the adipogenic differentiation program. We found that in addition to iAs^3+^ and iAs^5+^, trivalent methylated metabolites of iAs can also substantially suppress adipogenesis. We also determined that iAs^3+^, iAs^5+^, or MMA^3+^ attenuate and delay the DMI-induced reduction of CHOP10 protein expression at early stages of adipogenesis. The sustained expression of CHOP10 may sequester C/EBPβ and inhibit its transcriptional activation, thus suppressing adipogenesis.

iAs is metabolized by enzymatic and nonenzymatic mechanisms into MMA^3+^, MMA^5+^, DMA^3+^, and DMA^5+^ in humans. MMA (MMA^3+^ + MMA^5+^) and DMA (DMA^3+^ + DMA^5+^) are the major metabolites of iAs found in the human blood and urine (Pi et al. 2002). In addition, high levels of MMA and/or DMA were measured in the pancreas, liver, muscle, and WAT of mice with chronic iAs exposure (Paul et al. 2011). Therefore, even though adipocytes have low methylation capacity of iAs (Paul et al. 2007; Walton et al. 2004), adipose tissues may also be exposed to methylated arsenic species when humans or animals are under chronic iAs exposure. In the present study, we found that MMA^3+^ and DMA^3+^, but not MMA^5+^ and DMA^5+^, exhibited much more potent acute cytotoxicity and inhibitory effects on adipogenesis than iAs in 3T3-L1 cells, mouse ADSVFCs, and human ADSCs.

In the context of obesity and T2D, excess accumulation of WAT is a risk factor for insulin resistance and T2D. However, impairment of triglyceride storage in WAT also results in reduced insulin sensitivity (Vigouroux et al. 2011; Xue et al. 2012). For example, lipodystrophy—a syndrome in which adipogenesis is impaired and adipose tissue degeneration occurs—is associated with severe defects in lipid and glucose homeostasis (Garg and Agarwal 2009). Thus, maintaining a healthy fat function is essential for insulin sensitivity and metabolic homeostasis. Although the effect of iAs exposure on WAT development and function in humans is still unclear, emerging evidence has demonstrated that chronic iAs exposure is negatively associated with body mass index in adolescents and children (Su et al. 2012; Watanabe et al. 2007). iAs-exposed mice on a high-fat diet have been shown to accumulate less fat than unexposed control mice on the same diet (Paul et al. 2011). In contrast, the iAs-exposed mice exhibited glucose intolerance. Thus, chronic iAs exposure may cause WAT dysfunction, which is implicated in the development of insulin resistance and T2D.

UPR is a cellular stress response related to ER and is induced by the accumulation of unfolded protein aggregates or by excessive protein traffic (Banhegyi et al. 2007; Oyadomari and Mori 2004). Growing evidence shows that arsenic can activate UPR, which is initiated by inositol-requiring protein-1, PERK, and ATF6 (Oh et al. 2012). Among the genes that are transcriptionally regulated by ATF4, ATF6, and XBP1, CHOP10 is one of the mostly expressed inducible genes during ER stress (Oyadomari and Mori 2004). In addition to its critical role in regulating ER stress-mediated apoptosis, CHOP10 may dimerize with C/EBPβ and C/EBPα to serve as a transcriptional inhibitor in the regulation of adipogenesis (Ron and Habener 1992). CHOP10 that is expressed in growth-arrested preadipocytes transiently sequesters C/EBPβ by heterodimerization, and C/EBPβ is released when CHOP10 is down-regulated by the initiation of adipogenesis (Tang and Lane 2000). In the present study, we found that iAs and MMA^3+^ delayed and attenuated the DMI-induced reduction of CHOP10 by triggering UPR, which is correlated with their inhibitory effects on terminal adipogenesis. These findings suggest that arsenic may induce ER stress, trigger UPR, and interfere with adipose development and function.

Taken together, the present study demonstrates that low-level iAs and MMA^3+^ trigger UPR and induce CHOP10, a protein that inhibits C/EBPβ transcriptional activity, thus impairing adipogenesis. Given the importance of adipogenesis in adipose function and the evidence that various arsenicals suppress adipogenesis, future studies on the effect of chronic iAs exposure on adipogenesis and adipose development and function may provide novel insight into the diabetogenic effect of arsenic.

## Supplemental Material

(311 KB) PDF
