# Supplementary material for: Association between Arsenic Suppression of Adipogenesis and Induction of CHOP10 via the Endoplasmic Reticulum Stress Response
Source: Environ Health Perspect. 2012 Dec 5;121(2):237–43. doi: 10.1289/ehp.1205731 (PMC3569692; doi:10.1289/ehp.1205731)
Supplement: (311 KB) PDF [file ehp.1205731.s001.pdf]

Supplemental Material

# Association between Arsenic Suppression of Adipogenesis and Induction of CHOP10 via the Endoplasmic Reticulum Stress Response

Yongyong Hou, Peng Xue, Courtney G. Woods, Xia Wang, Jingqi Fu, Kathy Yarborough,

Weidong Qu, Qiang Zhang, Melvin E. Andersen, and Jingbo Pi

**Contents**

Methods ---------------------------------------------------------------------- Page 3

Table S1 ---------------------------------------------------------------------- Page 4

Figure S1 ---------------------------------------------------------------------- Page 5

Figure S2 ---------------------------------------------------------------------- Page 6

Figure S3 ---------------------------------------------------------------------- Page 7

Figure S4 ---------------------------------------------------------------------- Page 8

Figure S5 ---------------------------------------------------------------------- Page 9

Figure S6 ---------------------------------------------------------------------- Page 10

Figure S7 ---------------------------------------------------------------------- Page 11

Reference ---------------------------------------------------------------------- Page 11

## Methods

***Immunofluorescence microscopy.*** Briefly, 3T3-L1 preadipocytes were plated onto coverslips in 6-well plates and induced to differentiate using the DMI protocol 1 day after 100% confluence as achieved in the presence or absence of arsenic. At 16 h, cell monolayers were washed with cold phosphate buffered saline (PBS) and fixed for 10 min at room temperature in 2% (v/v) formaldehyde. After washing in PBS, cells were permeabilized in 1% (v/v) Triton X-100 in PBS, washed and incubated with 10% goat serum (sc-2043, Santa Cruz) in PBS for 1 h at room temperature. Cells were incubated with mouse monoclonal $IgG_{2a}$ against C/EBPβ (sc-7962; 1:500, Santa Cruz) in 10% goat serum in PBS for 16 h at 4°C and subsequently with Rhodamine-linked goat-anti-mouse IgG-R (sc-2092, 1:50, Santa Cruz) for 45 min at room temperature. After PBS washing, the coverslips were mounted with the Prolong Gold antifade reagent with DAPI (P36931, Invitrogen) onto microscope slides and examined using an Axio Observer Z1 fluorescence microscope (Carl Zeiss, Inc. Oberkochen, Germany). Image analysis on the number and intensity of fluorescent aggregation was performed using a wavelet-based, Microsoft Windows-compatible computer program developed by Larry S. Barak at Duke University (Kapur et al. 2009).

## Table S1. Primer sequences for Real-time RT-qPCR

| Abbreviations | Gene name | Forward (5' - 3') | Reverse (5' - 3') |
|---|---|---|---|
| *18S* | mouse 18S ribosomal RNA | CGAACGTCTGCCCTATCAACTT | CCGGAATCGAACCCTGATT |
| *Adrp* | mouse Adipose differentiation-related protein | GGTGATGGCAGGCGACAT | CCATCGGACACTTCCTTAAAGG |
| *Atf4* | mouse Activating transcription factor 4 | CTCAGACAGTGAACCCAATTGG | GGCAACCTGGTCGACTTTTATT |
| Adiposin | mouse Adiposin | GCTATCCCAGAATGCCTCGTT | TTCCACTTCTTTGTCCTCGTATTG |
| *Cd36* | mouse Cluster of differentiation 36 | CAGAGTTCGTTATCTAGCCAAGGAA | CATTGGGCTGTACAAAAGACACA |
| *Cebpα* | mouse CCAAT-enhancer-binding protein α | CGCAAGAGCCGAGATAAAGC | CGGTCATTGTCACTGGTCAACT |
| *Cebpβ* | mouse CCAAT-enhancer-binding protein β | AAGCTGAGCGACGAGTACAAGA | GTCAGCTCCAGCACCTTGTG |
| *Cebpδ* | mouse CCAAT-enhancer-binding protein δ | GCCGTGCCCACCCTAGA | CGCTTTGTGGTTGCTGTTGA |
| *Cebp* ε | mouse CCAAT/enhancer binding protein ε | CGGCCATTCGCCTATCC | GCCCCAAAGCCTTCCTATCT |
| *Chop10* | mouse C/EBP homologous protein | CCTTTATTCTGCGGAGCTGAA | AGGGCCACAGAAGGATGGA |
| *Gadd45a* | mouse growth arrest and DNA-damage-inducible 45 α | GACGACGACCGGGATGTG | AGCAGAACGCACGGATGAG |
| *Lpl* | mouse lipoprotein lipase | GGACTGAGGATGGCAAGCAA | GCCACTGTGCCGTACAGAGA |
| *Pparγ1* | mouse Peroxisome proliferator-activated receptor γ1 | GGGCTGAGGAGAAGTCACAC | TGGTTCACCGCTTCTTTCA |
| *Pparγ2* | mouse Peroxisome proliferator-activated receptor γ2 | TGCTGTTATGGGTGAAACTCTG | CTGTGTCAACCATGGTAATTTCTT |
| *Rxr* α | mouse retinoid X receptor α | GCCGGCCTCTGACTGTGA | GCACCACAATGTCCCAGTGA |
| *Srebp1* | mouse Sterol regulatory element-binding protein 1 | AGCCCACAATGCCATTGAG | CAGGTCTTTGAGCTCCACAATCT |
| *sXbp1* | mouse spliced X-box binding protein 1 | GAGTCCGCAGCAGGTG | GTGTCAGAGTCCATGGGA |
| *Xbp1* | mouse X-box binding protein 1 | ACGGCCTTGTGGTTGAGAAC | AGGATCCAGCGTGTCCATTC |
| *LPL* | human lipoprotein lipase | TCCGCGTGATTGCAGAGA | GCTCGTGGGAGCACTTCACT |
| *CD36* | human Cluster of differentiation 36 | AGAGTTCGTTTTCTAGCCAAGGAA | TTGGGCTGCAGGAAAGAGACT |
| *PPAR1* | human Peroxisome proliferator-activated receptor γ1 | GGCCGCAGATTTGAAAGAAG | TCGTTTGAGAAAATGGCCTTGT |
| *PPAR2* | human Peroxisome proliferator-activated receptor γ2 | CAAACCCCTATTCCATGCTGTT | GCTTTCTGGGTCAATAGGAGAATC |
| *18S* | human 18S ribosomal RNA | CGCCCCAGCACTTTGG | TTACCAGCGGATGGATGGA |

**DMI protocol for 3T3-L1 cells:**

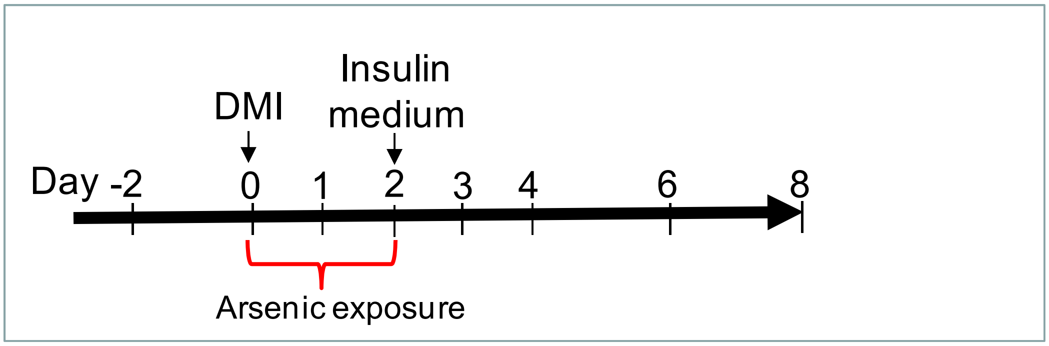


**DMIRI protocol for mouse ADSVFCs and human ADSCs:**

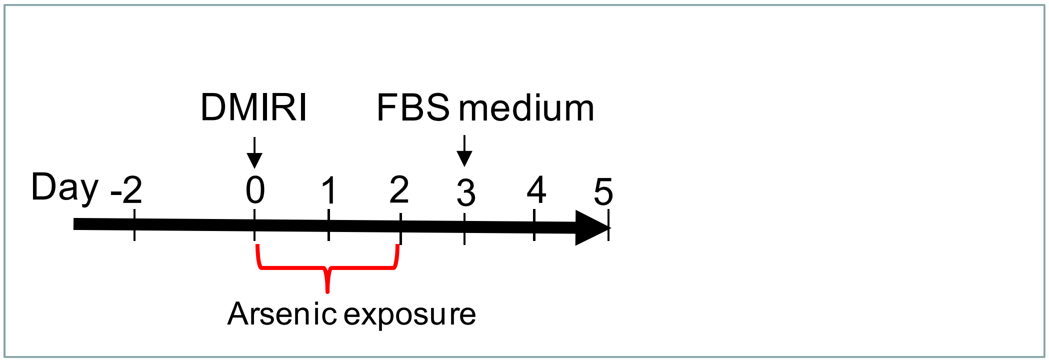


**Figure S1.** Protocols for adipogenic differentiation and arsenic exposure in 3T3-L1 cells, mouse ADSVFCs and human ADSCs. Cells were differentiated 1 day after confluence (designated as day 0) by replacing growth medium with differentiation medium as indicated (Black arrows). DMI: 1 μM dexamethasone, 0.5 mM IBMX and 1 μg/ml insulin in DMEM with 10% FBS; Insulin medium, 1 μg/ml insulin in DMEM with 10% FBS; DMIRI: DMI + 1 μM rosiglitazone and 125 μM indomethacin in DMEM with 10% FBS; FBS medium, DMEM with 10% FBS. Arsenicals were added during the first two days of adipogenic differentiation.

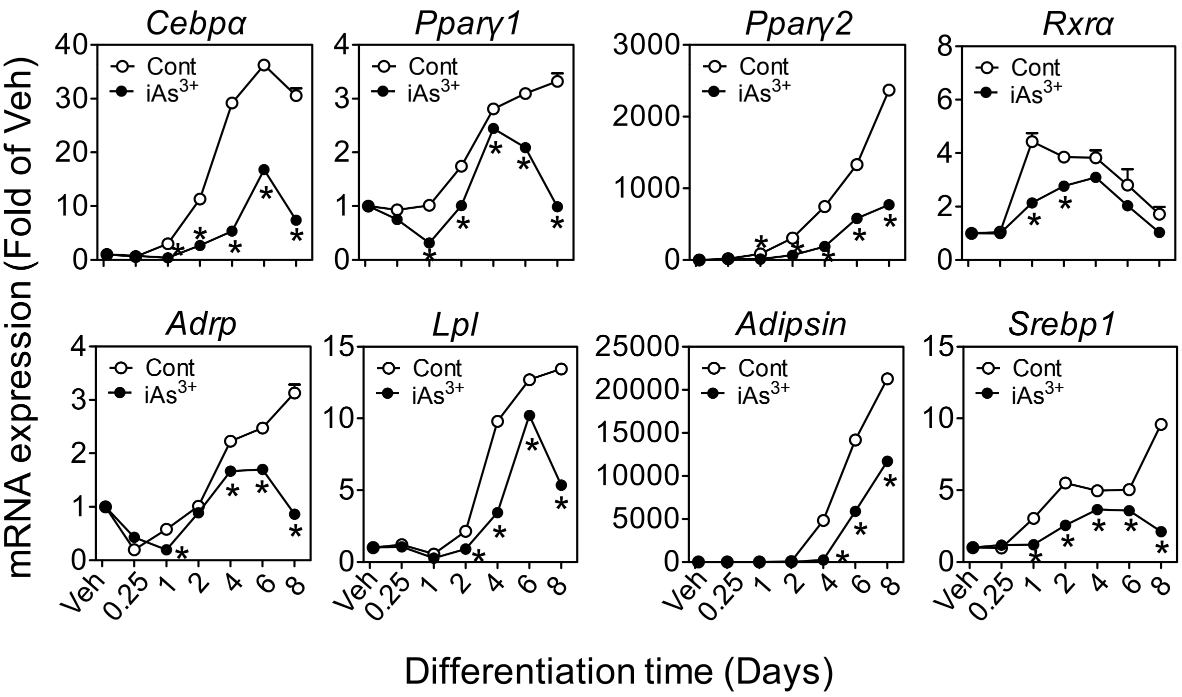


**Figure S2.** Inhibitory effect of $iAs^{3+}$ exposure on mRNA expression of adipogenic genes during adipogenesis induced by hormonal cocktail DMI. Cells were differentiated with DMI protocol alone (Control, Cont) or with 5 μM $iAs^{3+}$ added during the first 48 h of differentiation. Vehicle (Veh) indicates the 3T3-L1 cells without DMI treatment, * $p < 0.05$ $iAs^{3+}$-treated vs. Cont with the same treatment.

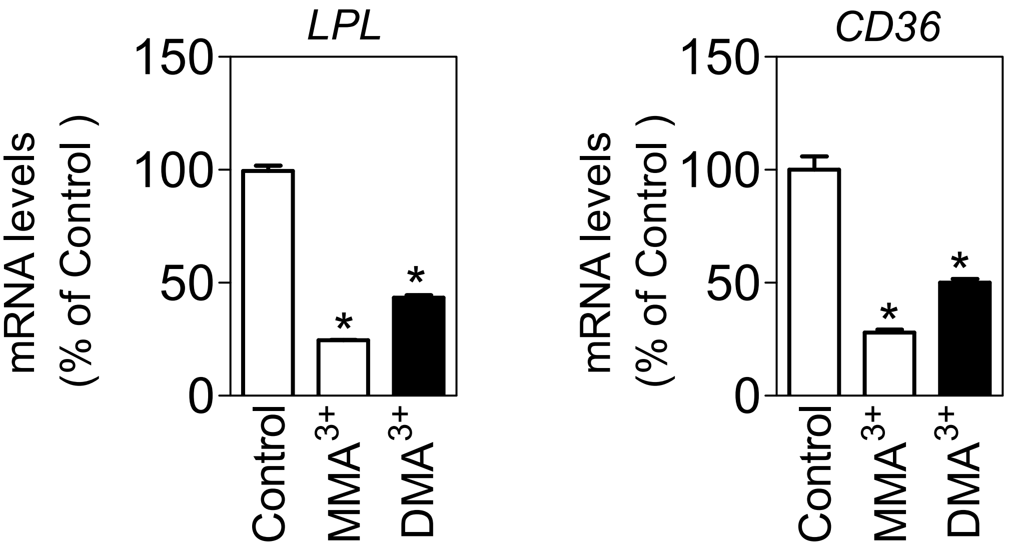


**Figure S3.** $MMA^{3+}$ and $DMA^{3+}$ suppress adipogenic gene expression in human ADSCs. The cells were cultured to 95% confluence and differentiated for 5 days using DMIRI protocol, followed by an immediate measurement of mRNA expression. DMIRI protocol, cells were differentiated by replacing growth medium with DMIRI differentiation medium containing 1 μM dexamethasone, 0.5 mM IBMX, 1 μg/ml insulin, 1 μM rosiglitazone and 125 μM indomethacin in DMEM with 10% FBS. After three days the medium was changed to DMEM with 10% FBS and maintained for 2 additional days. $MMA^{3+}$ (0.2 μM) or $DMA^{3+}$ (2 μM) was added during Day 1 and 2 of differentiation. * $p < 0.05$ Arsenic-treated vs. Control.

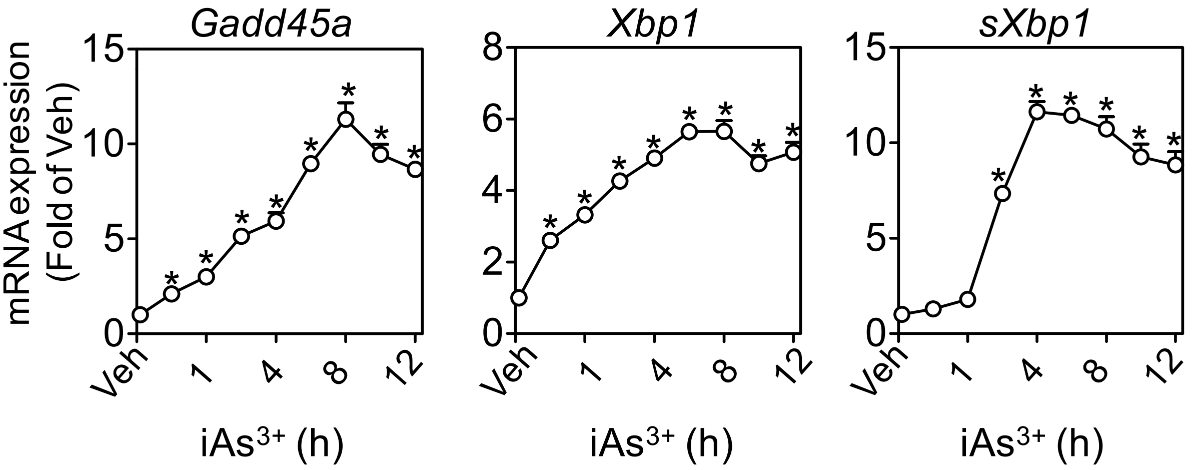


**Figure S4.** Effect of $iAs^{3+}$ on the mRNA expression of UPR genes in 3T3-L1 cells. Cells were exposed to 5 μM $iAs^{3+}$ in DMEM with 10% CS for the indicated time. n = 3. * $p < 0.05$ $iAs^{3+}$-treated vs. Vehicle (Veh).

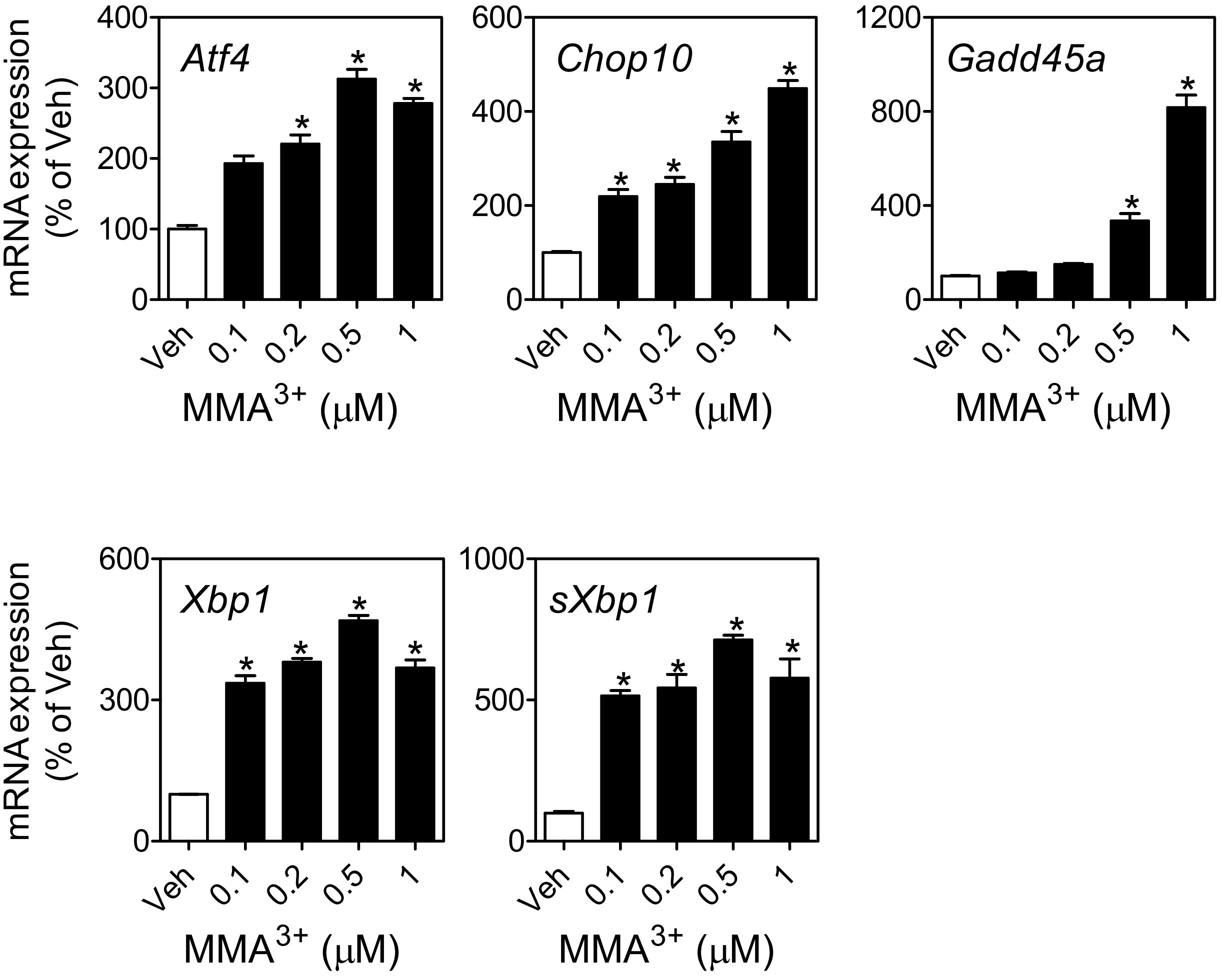


**Figure S5.** Effect of $MMA^{3+}$ on the mRNA expression of UPR genes in 3T3-L1 cells. Cells were exposed to $MMA^{3+}$ in DMEM with 10% CS for 6 h. n = 3. * $p < 0.05$ $MMA^{3+}$-treated vs. Vehicle (Veh).

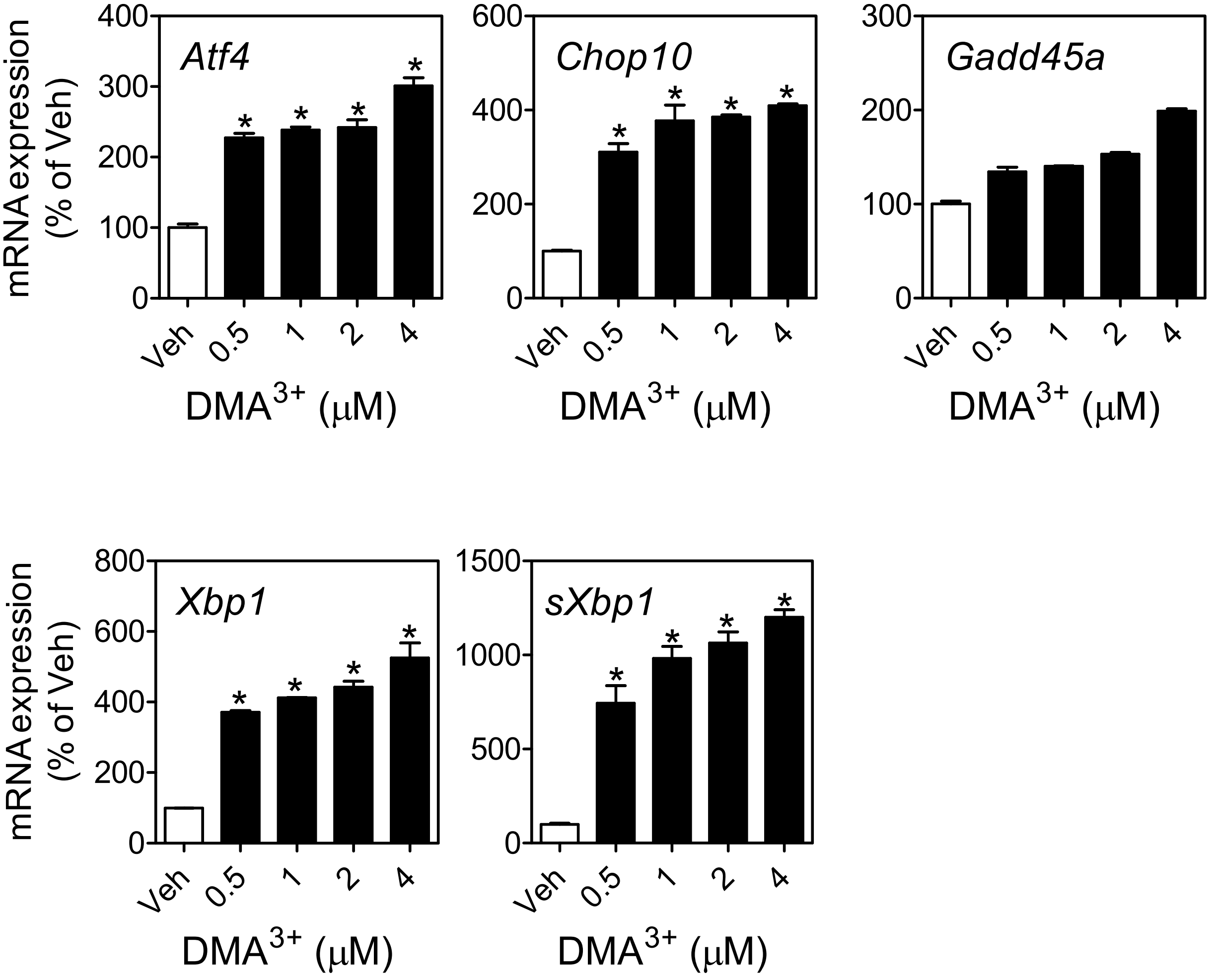


**Figure S6.** Effect of $DMA^{3+}$ on the mRNA expression of UPR genes in 3T3-L1 cells. Cells were exposed to $DMA^{3+}$ in DMEM with 10% CS for 6 h. n = 3. * $p < 0.05$ $DMA^{3+}$-treated vs. Vehicle (Veh).

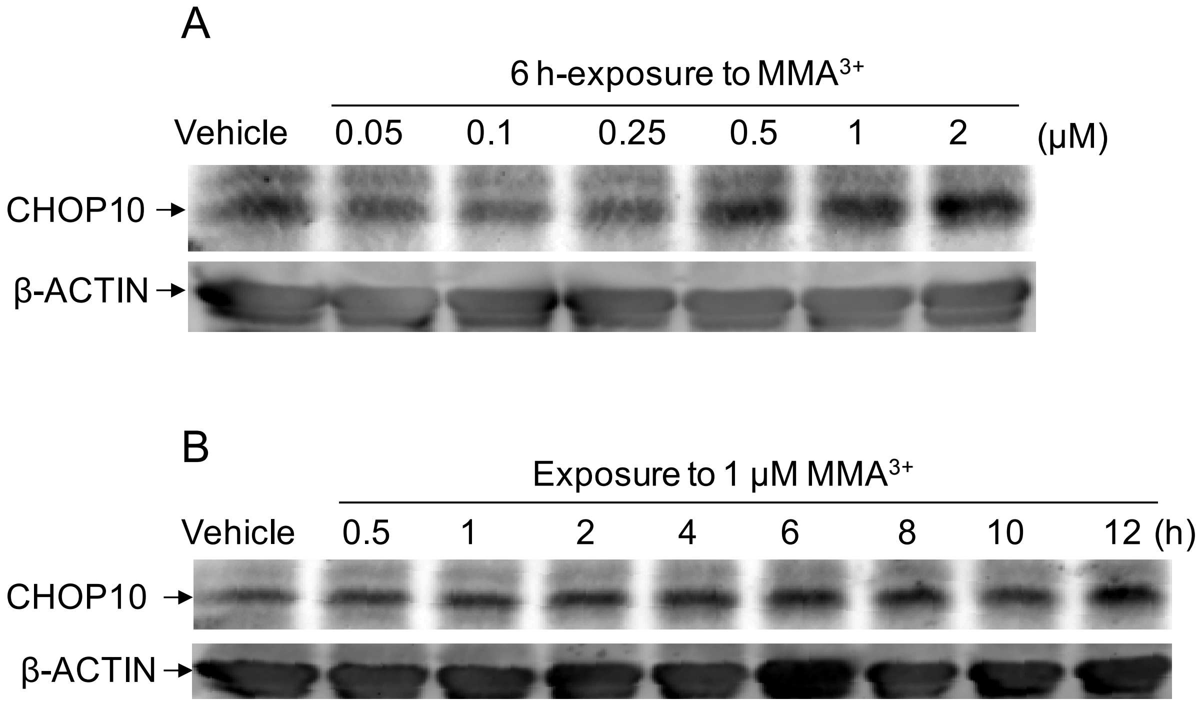


**Figure S7.** Concentration-response (A) and time course (B) of $MMA^{3+}$-induced protein expression of CHOP10 in 3T3-L1 preadipocytes. Cells were exposed to $MMA^{3+}$ in DMEM with 10% CS.

**References:**

Kapur A, Zhao P, Sharir H, Bai Y, Caron MG, Barak LS, et al. 2009. Atypical responsiveness of the orphan receptor GPR55 to cannabinoid ligands. J Biol Chem 284(43): 29817-29827.
